# A Simultaneous Multiparametric ^18^F-FDG PET/MRI Radiomics Model for the Diagnosis of Triple Negative Breast Cancer

**DOI:** 10.3390/cancers14163944

**Published:** 2022-08-16

**Authors:** Valeria Romeo, Panagiotis Kapetas, Paola Clauser, Pascal A. T. Baltzer, Sazan Rasul, Peter Gibbs, Marcus Hacker, Ramona Woitek, Katja Pinker, Thomas H. Helbich

**Affiliations:** 1 Department of Advanced Biomedical Sciences, University of Naples Federico II, Via S. Pansini 5, 80138 Naples, Italy; 2 Division of General and Pediatric Radiology, Department of Biomedical Imaging and Image-Guided Therapy, Medical University of Vienna, Waehringer Guertel 18-20, 1090 Wien, Austria; 3 Division of Nuclear Medicine, Department of Biomedical Imaging and Image-Guided Therapy, Medical University of Vienna, Waehringer Guertel 18-20, 1090 Wien, Austria; 4 Breast Imaging Service, Department of Radiology, Memorial Sloan Kettering Cancer Center, 300 E 66th Street, New York, NY 10065, USA; 5 Department of Radiology, University of Cambridge, Cambridge CB2 0QQ, UK

**Keywords:** ^18^F-FDG PET/MRI, breast cancer, machine learning, artificial intelligence

## Abstract

**Simple Summary:**

In this study, we aimed to build a machine-learning predictive model for the identification of triple negative breast cancer, the most aggressive subtype, using quantitative parameters and radiomics features extracted from tumor lesions on hybrid PET/MRI. The good performance of the model supports the hypothesis that hybrid PET/MRI can provide quantitative data able to non-invasively detect tumor biological characteristics using artificial intelligence software and further encourages the conduction of additional studies for this purpose.

**Abstract:**

Purpose: To investigate whether a machine learning (ML)-based radiomics model applied to ^18^F-FDG PET/MRI is effective in molecular subtyping of breast cancer (BC) and specifically in discriminating triple negative (TN) from other molecular subtypes of BC. Methods: Eighty-six patients with 98 BC lesions (Luminal A = 10, Luminal B = 51, HER2+ = 12, TN = 25) were included and underwent simultaneous ^18^F-FDG PET/MRI of the breast. A 3D segmentation of BC lesion was performed on T2w, DCE, DWI and PET images. Quantitative diffusion and metabolic parameters were calculated and radiomics features extracted. Data were selected using the LASSO regression and used by a fine gaussian support vector machine (SVM) classifier with a 5-fold cross validation for identification of TNBC lesions. Results: Eight radiomics models were built based on different combinations of quantitative parameters and/or radiomic features. The best performance (AUROC 0.887, accuracy 82.8%, sensitivity 79.7%, specificity 86%, PPV 85.3%, NPV 80.8%) was found for the model combining first order, neighborhood gray level dependence matrix and size zone matrix-based radiomics features extracted from ADC and PET images. Conclusion: A ML-based radiomics model applied to ^18^F-FDG PET/MRI is able to non-invasively discriminate TNBC lesions from other BC molecular subtypes with high accuracy. In a future perspective, a “virtual biopsy” might be performed with radiomics signatures.

## 1. Introduction

Breast cancer (BC) is a heterogeneous disease with a multifactorial etiology (e.g., hormone, genetics-related) affecting the capability of cells to repair DNA damages [1,2,3]. In the course of cancer development, cells progressively accumulate mutations, and acquire new cancer hallmark capabilities, i.e., sustaining proliferative signaling, evading growth suppressors, resisting cell death, enabling replicative immortality, inducing/accessing vasculature, activating invasion and metastasis, reprogramming cellular metabolism, and avoiding immune destruction [4,5]. Depending on the expression of molecular biomarkers, including estrogen receptor (ER), progesterone receptor (PgR), and human epidermal growth factor receptor 2 (HER2), BC can be categorized into different subtypes [6]. The knowledge of such molecular features led to the development of targeted treatments and improved outcomes [7]. An exception is the triple negative (TN) BC, which does not express any of these molecular biomarkers. TN is the most aggressive BC subtype, with a propensity for tissue invasion and distant metastases [8], and has the poorest prognosis as no targeted treatment is currently available [9].

The assessment of molecular subtypes is, therefore, the “sine qua non” for treatment planning of BC. In patients with TNBC, it is especially important as they usually require upfront systemic treatment before surgery. Currently, the assessment of molecular subtypes is performed through invasive tissue sampling using invasive core needle biopsy, an approach inherently limited by sampling bias and providing only a snapshot of the biology of the entire tumor. Indeed, molecular biomarkers are likely to differ within the tumor, a phenomenon known as intratumor heterogeneity [10]. The development of a non-invasive, pre-operative approach for the molecular characterization of BC in its entirety that can inform whether a tumor really has no actionable treatment targets, i.e., is TNBC entirely, is still an unmet clinical need.

Developments have led to sophisticated imaging techniques that can depict the functional properties of breast tumors and their underlying biology. Currently, ^18^F-fluorodeoxyglucose positron emission tomography (^18^F-FDG PET) as well as magnetic resonance imaging (MRI) diffusion-weighted imaging (DWI), and dynamic contrast-enhanced (DCE) techniques are the gold standard for the in vivo assessment of tumor metabolism (^18^F-FDG PET), cellularity (DWI) and neoangiogenesis (DCE) [11,12,13]. For the assessment of TNBC in particular, both ^18^F-FDG PET and MRI have shown high sensitivity [14,15]. More recently, simultaneous ^18^F-FDG PET/MRI has been shown to be promising for the accurate and non-invasive biological characterization of BC [16].

Initial studies have demonstrated the potential of radiomics analysis coupled with machine learning (ML) based on either PET or MRI (both DCE and DWI) for the comprehensive assessment of tumor phenotypes and for the development of predictive models [17,18,19]. Most recently, the value for artificial intelligence (AI)—enhanced simultaneous ^18^F-FDG PET/MRI for BC phenotyping, specifically for hormone receptor-positive (luminal) BC identification has been demonstrated [20]; nevertheless, its potential for the identification of TNBC remains unclear.

We hypothesized that AI-enhanced simultaneous ^18^F-FDG PET/MRI can identify the radiomic signature of TNBC. Therefore, we aimed to build an ML-based predictive model using both quantitative imaging parameters and radiomic features extracted from simultaneous ^18^F-FDG PET/MRI to distinguish TNBC from other molecular BC subtypes.

## 2. Materials and Methods

### 2.1. Patient Sample

This prospective single-institution study was approved by the institutional review board, and written informed consent was obtained from all participants. All patients underwent simultaneous multiparametric ^18^F-FDG PET/MRI of the breast between June 2016 and June 2020. Inclusion criteria were: >18 years-old subjects; histologically verified BC lesions; and not pregnant or breastfeeding. Exclusion criteria were: patients with standard contraindications for performing MRI examinations (e.g., metal implants, metallic foreign bodies, renal failure with eGFR < 30 mL/min); patients for whom histological proof of malignancy was not available; patients with malignant lesions other than BC; tumor recurrence; incomplete ^18^F-FDG PET/MRI examinations; and patients with PET, DCE, or DWI images that were not suitable for subsequent multiparametric and radiomics analyses. Patients included in this study have been investigated in a previous study with different purpose and results [21].

### 2.2. ^18^F-FDG PET/MRI Acquisition Protocol

Simultaneous ^18^F-FDG PET/MRI was performed as previously described [21], using a Biograph mMR system (Siemens Healthineers, Erlangen, Germany), which is a hybrid system combining an MRI-compatible PET detector with a 3.0 Tesla MRI scanner. During PET data acquisition, T2-weighted, diffusion tensor imaging (DTI) single-shot spin-echo-prepared echo-planar imaging (EPI) sequence and high temporal resolution DCE-MRI sequences were acquired. Details of ^18^F-FDG PET/MRI protocol are reported in Supplementary Materials S1 [22,23,24,25].

### 2.3. Image Analysis

#### Quantitative Parameters

A board-certified breast radiologist and a nuclear medicine physician with 7 and 11 years of experience, respectively, independently evaluated all PET/MR images, using a previously described method that proved to be highly reproducible [21].

MR images were analyzed for both DWI and perfusion-weighted imaging (PWI) quantitative parameters using a free, open-source software (Horos v.3.3.5, distributed under the LGPL license at Horosproject.org, sponsored by Nimble Co LLC d/b/a Purview in Annapolis, MD, USA). In detail, 2D circle ROIs were placed on apparent diffusion coefficient (ADC) maps for ADCmean calculation of tumor lesion and contralateral breast parenchyma. Thereafter, 2D ROIs were drawn over tumor lesions on first post-contrast DCE images and then pasted on perfusion maps DCE maps for the extraction of quantitative perfusion parameters, including mean transit time (MTT), plasma flow (PF), and volume distribution (VD), according to previous evidence [26]. Details of DWI and DCE image analysis are reported in Supplementary Materials S2.

PET images were analyzed for the quantification of tumor uptake using the Hermes Hybrid Viewer (Hermes Medical Solutions, Stockholm, Sweden). Maximum, mean, and minimum standardized uptake values (SUVmax, SUVmean, and SUVmin) were calculated by placing a 3D volume of interest (VOI) with a fixed threshold at the level of tumor lesions; care was taken to exclude surrounding background parenchymal uptake. The same approach was used for the extraction of SUVmean of the ipsilateral and contralateral normal appearing breast parenchyma, away from the nipple and areola.

### 2.4. Radiomics Analysis

#### 2.4.1. Tumor Segmentation

**Figure 1:**
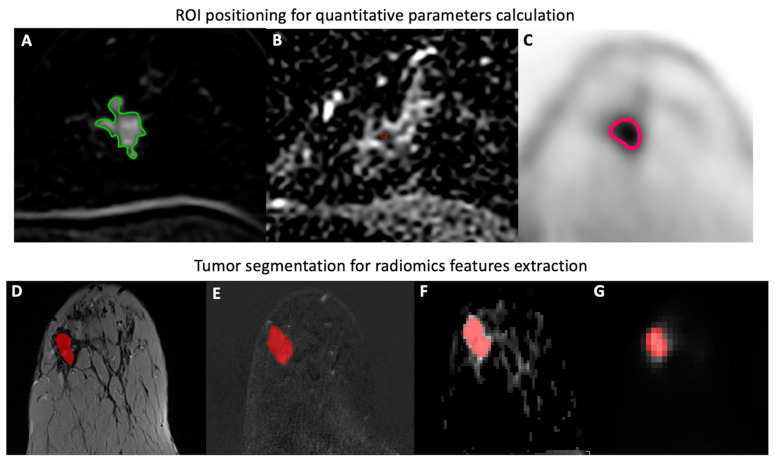
Examples of 2D ROI placement for the extraction of quantitative parameters (mean transit time; plasma flow; volume distribution; ADC mean; and SUVmax, mean, and minimum) (**A**–**C**), and whole tumor segmentation for radiomics features (first, second, and higher order) extraction (**D**–**G**) from primary BC tumor lesions on DCE (**A**,**E**), DWI (**B**,**F**), PET (**C**,**G**), and T2-weighted (**D**) images.

Whole BC lesions were segmented on T2-weighted, DCE, DWI, and PET images using a dedicated software (ITK-SNAP v. 3.6.0, itksnap.org, University of Pennsylvania, Philadelphia, PA, USA; University of Utah, Salt Lake City, UT, USA). DCE (first post-contrast timepoint), DWI, and PET images were annotated using a semi-automated method selecting a lower boundary of signal intensity, while a slice-by-slice approach was used for segmenting BC lesions on T2-weighted images. In all cases, VOIs were placed within the margins of the lesions and care was taken to exclude macroscopic necrosis as well as cystic and hemorrhagic areas or biopsy markers. Figure 1 illustrates ROI placement on DWI, PWI and PET images, as well as the BC lesion segmentation process.

#### 2.4.2. Radiomic Feature Extraction

Prior to the extraction of radiomic features, data for all images were reduced to 16 grey levels. The Computational Environment for Radiological Research (CERR), compatible with the Image Biomarker Standardization Initiative (IBSI), platform was used to extract radiomic features [27] from DCE, T2-weighted, ADC and PET images. For the extraction of ADC-derived radiomic features, BC segmentation was first performed on DWI images to better define tumor margins and leverage their intrinsic high contrast, and ROIs on DWI images were subsequently pasted onto ADC maps for the calculation of radiomic features. For non-isotropic images (T2-weighted images and ADC maps), feature extraction was performed in a slice-by-slice 2D fashion and successively clustered over the whole lesion (BTW3 as defined by IBSI) [28]. Due to the large class imbalance present, adaptive synthetic sampling was utilized to remove this effect. A total of 101 features were computed per image; all features are detailed in Supplementary Materials S3.

#### 2.4.3. Radiomic Feature Selection and Machine Learning

As our study dataset consisted of a limited number of cases relative to a high number of extracted features, radiomic feature selection was performed using Least Absolute Shrinkage and Selection Operator (LASSO) regression [29], and subsequently, the five most important features were selected for each radiomic model to avoid overfitting. With insufficient cases to fine tune the LASSO hyperparameter (Lambda, a pragmatic approach was taken, wherein the algorithm employed automatically selected the largest value of Lambda that resulted in a nonnull model. Thereafter, a fine Gaussian support vector machine (SVM) was employed for radiomic model building using MATLAB 2017b (The MathWorks Inc., Natick, MA, USA). SVM is a widely used supervised ML method that has demonstrated good performance in small datasets and also provides memory efficient models that are able to solve both linear and non-linear issues [30]. Briefly, SVM works by identifying a hyperplane that best segregates two classes (e.g., TNBC vs. other BC subtypes). The choice of the best hyperplane is made based on how many cases are correctly classified and with which margins. The higher the margin, the higher the robustness of the model reducing the possibility of misclassification. Due to the limited number of cases in our study dataset, it was not possible to define a training and a validation set. Therefore, five-fold cross validation was employed, wherein five groups of data were generated, so that each model was trained on the first four groups and tested on the remaining one, providing reliable information on model generalizability. Data were initially standardized (z-score calculation with mean 0 and standard deviation 1) to prevent dependence on any individual parameter, especially those parameters containing high values. The whole process was repeated 1000 times, for each of the 8 datasets, to provide final accuracy metrics. Different models were built using various combinations of DCE, T2-weighted, ADC, and PET-derived radiomic features as well as quantitative PET/MRI parameters, to evaluate their ability to accurately distinguish TNBC from other BC subtypes.

### 2.5. Reference Standard

Malignant tumor samples from core biopsy and/or surgical specimen were analyzed to define tumor histology, grade, and immunohistochemical status including ER, PgR, Ki-67 expression, and overexpression and/or amplification of HER2 of each breast cancer lesion. The St. Gallen surrogate molecular subtype definitions were used to classify breast lesions [31].

### 2.6. Statistical Analysis

The Kolmogorov–Smirnov test was performed to assess whether data were normally distributed. Accordingly, the Mann–Whitney test or independent *t*-test were performed to assess differences in terms of lesion size, quantitative parameters and radiomics parameters between TN and non-TN breast cancer subtypes. McNemar’s test was used to assess differences in terms of diagnostic performance among the different radiomics models. *p* values ≤ 0.05 were considered statistically significant. Confidence intervals for diagnostic metrics were calculated using a bootstrapping approach. Statistical analysis was conducted using SPSS, Version 25.0. 2017 (IBM Corp, Armonk, NY, USA).

## 3. Results

### 3.1. Patient Sample

**Figure 2:**
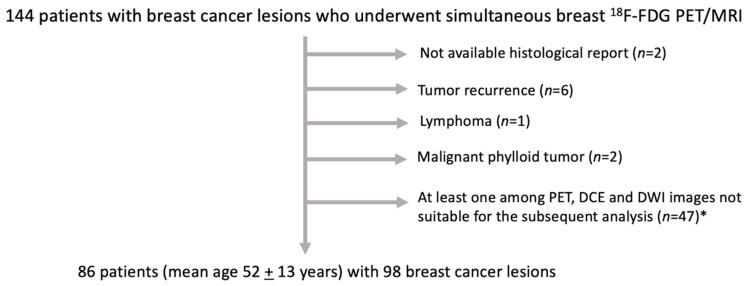
Flowchart of patient selection. * Low quality images, DCE images for which maps calculation was not feasible, small breast cancer lesions.

According to the inclusion and exclusion criteria, 144 patients were initially enrolled in the study. Of these, 86 female patients (mean age 52 ± 13 years) were included in the final study sample, with 98 histologically proven BC lesions (mean size: 28.31 ± 16.8 mm), comprising 25 TN, 10 Luminal A, 51 Luminal B, and 12 HER2+ lesions. In detail, there were eight patients with two BC lesions in the same breast and four patients with bilateral BC lesions. The majority (80%, 79/98) of histological types was represented by ductal invasive carcinoma. The flowchart of patient inclusion is illustrated in Figure 2, while histological details of included BC lesions are reported in Supplementary Materials S4.

TN lesions showed significantly higher SUVmax (9.5 vs. 4.9), SUVmean (5.7 vs. 3.4), and SUVmin (3.1 vs. 2) compared with lesions of other BC subtypes (*p* < 0.001). No differences were observed in terms of lesion size and remaining quantitative parameters between TN and non-TN lesions. Mean values of tumor size and quantitative parameters are reported in Supplementary Materials S5. The single best performing quantitative parameter was SUVmax with an AUROC of 0.83 (95% CI: 0.76–0.90).

### 3.2. Feature Selection and Machine Learning Analysis

Eight radiomic models (Models 1–8) were developed for TNBC identification, using the following combinations of quantitative parameters and radiomic features: quantitative parameters alone (Model 1); radiomic features extracted from ADC (Model 2), DCE (Model 3), PET (Model 4) and T2-weighted (Model 5) images; combinations of radiomic features extracted from different ^18^F-FDG PET/MR images, namely DCE-MRI and ADC (Model 6), and DCE-MRI, ADC, and PET (Model 7); and quantitative parameters combined with radiomic features (Model 8). Quantitative parameters and/or radiomic features selected for each model are reported in Table 1.

Across all models, the area under the receiver operating curve (AUC) ranged from 0.725 (Model 5, 95% CI: 0.679–0.765) to 0.887 (Model 7, 95% CI: 0.847–0.916). Model 7 showed the best performance in discriminating TNBC from other BC subtypes, with a diagnostic accuracy of 82.8% (95% CI: 78.2–87.1%), sensitivity of 79.7% (95% CI: 71.6–86.5%), and specificity of 86.0% (95% CI: 80.8–90.4%). In detail, Model 7 included size zone matrix-based features extracted from ADC and PET images as well as first-order and neighborhood gray level dependence matrix-based features extracted from PET images. Of note, Model 1, based on quantitative parameters extracted from both primary lesions (SUVmax, PF, MTT, ADCmean) and contralateral breast tissue (ADCmean), achieved the second-best AUC of 0.884 (95% CI: 0.867–0.898), followed by Model 8, combining quantitative parameters and radiomic features, which showed an AUC of 0.871 (95% CI: 0.849–0.889).

Among the models built using radiomic features extracted from individual ^18^F-FDG PET/MR images, the best performing one was Model 2, based on features extracted from ADC maps, which yielded an AUC of 0.826 (95% CI: 0.758–0.984). On the other hand, the worst performing one was Model 5, based on features extracted from T2-weighted images, which yielded an AUC of 0.725 (95% CI: 0.679–0.765). Accuracy metrics of all radiomic model are reported in Table 2.

A statistically significant difference in terms of diagnostic performance was found using McNemar’s test between the best performing model (Model 7) and the worst performing one (Model 5) (*p* = 0.005). No differences were observed between Model 7 and the remaining radiomic models. Comparisons in terms of diagnostic performance among all models are reported in Supplementary Materials S6, while univariable results for the image features and quantitative parameters are presented in Supplementary Materials S7.

## 4. Discussion

In the present study, we evaluated AI-enhanced simultaneous ^18^F-FDG PET/MRI to distinguish TNBC from other molecular BC subtypes. To this end, we built ML-based predictive models employing radiomic features and/or quantitative parameters extracted from simultaneous ^18^F-FDG PET/MRI to non-invasively identify TNBC. Model 7, the best performing one (AUC of 0.887; 95% CI: 0.847–0.916) specifically included features extracted from functional images i.e., ADC and PET, supporting the hypothesis that functional data such as tumor cellularity and metabolism may better depict biological tumor features compared to morphologic sequences. It is worth noting that no significant differences in terms of diagnostic performance were found between Model 7 and all other models except for Model 5 which was based on solely on radiomic features extracted from T2-weighted images.

Such findings support the expectation that, in the near future, molecular data could be non-invasively obtained by imaging through the application of artificial intelligence tools. This issue is particularly relevant if we consider that ^18^F-FDG PET and MRI are already indicated for both local and global staging of locally advanced breast cancer as well as for treatment monitoring. As such, it is permissible to imagine that, with a single imaging examination, tumor diagnosis, staging, and phenotyping could be obtained non-invasively at the same time. In this light, “virtual biopsies” could be performed once radiomic profiles specific to molecular subtypes have been defined, aiming at providing genetic and phenotypic alterations which are representative of the whole tumor and comprehensively describe tumor heterogeneity. Furthermore, the extraction of quantitative imaging data from the whole tumor could allow the spatio-longitudinal monitoring of biomarker heterogeneity changes during treatment and the early identification of clonal dynamics and genetic modifications related to the occurrence of drug resistance.

PET and MRI represent the most promising imaging modalities for this purpose, due to their ability to non-invasively inform on cancer metabolism, cellularity, and neoangiogenesis. Such properties are reported to be different between BC subtypes which exhibit different biological aggressiveness and behavior [32]. TNBC is characterized by higher glucose metabolism, which is reflected in its higher SUV values compared with other BC subtypes as seen in other studies [32,33] as well as in this investigation. However, quantitative parameters (e.g., DWI-ADC values) alone do not seem to be accurate enough to discriminate molecular BC subtype; thus, more sophisticated AI-enhanced approaches are necessary [34].

So far, several attempts have been made to non-invasively define BC molecular subtype through the use of radiomics and machine learning applied to PET and MRI [17,18,19]. Previous studies explored the feasibility and usefulness of radiomics applied to PET/CT or MRI (both DCE and DWI) features for the prediction of molecular subtype [17,18,19,20,35]. Only recently have radiomics and AI techniques been applied to simultaneous ^18^F-FDG PET/MRI for a comprehensive analysis of molecular subtype, Ki67 expression, nodal status, and presence of distant metastasis in a population of 124 BC patients [20]. For the prediction of molecular BC subtypes, Umutlu et al. built two accurate radiomic models to discriminate Luminal A vs. Luminal B BC (AUC: 0.978, 95% CI: 0.950–1.000) and Luminal BC vs. other subtypes (AUC: 0.950, 95% CI: 0.922–0.979), based on MRI and PET-derived radiomics features, respectively. In contrast, our best performing radiomics model included radiomic features extracted from both MRI and PET images; furthermore, our model was geared towards specifically identifying TNBC. This choice was driven by the particularly aggressive biological behavior of TNBC and the different biological development of TNBC compared with other BC subtypes, supposed to originate from luminal progenitors and breast epithelial stem cells, respectively, according to a cell-of-origin hypothesis [36]. However, according to a more recent hypothesis, Luminal and TNBC could have a common luminal progenitor and the latter, through a dedifferentiation process, could acquire a basal like phenotype [37]. Furthermore, both types of tumor cells are supposed to be present in the same BC lesions, even if in different percentages, which also contributes to both intra- and intertumor heterogeneity [6]. Others have attempted to diagnose TNBC using radiomic signatures extracted from 300 pre-treatment post-contrast CT examinations [38]. Five radiomic features were selected, showing AUC values of 0.881 (95% CI: 0.781–0.921) and 0.851 (95% CI: 0.761–0.961) in the training and validation group, respectively. While this single study yielded comparable results, it has to be noted that CT is not an imaging modality that is recommended for breast imaging and has no clinical standing in breast cancer diagnosis and treatment monitoring.

Our study has several limitations to be acknowledged, the first being the relatively small sample size, as accessibility to simultaneous ^18^F-FDG PET/MRI was limited due to high demand from clinical needs. Furthermore, TNBC is relatively rare compared to other subtypes, thus larger numbers can only be recruited in a multi-centric setting over a reasonable time period. Due to this limitation, we refrained from using a subset of cases as a held-out test set. Indeed, a five-fold cross-validation was used, as done in previous studies involving the preliminary assessment of the applicability of the model to an unseen population [21,39]. Potentially, fewer parameters for each model might have been preferable but, considering the 25 cases included in the minority class, this equates to 5 cases per feature, which was deemed acceptable. A known limitation of LASSO is that in case of highly correlated features, the selection of features among those that are correlated can be random, or at least noisy. By employing LASSO in a cross-validated fashion, this effect is reduced to a certain extent. However, it is noted that rerunning the LASSO process may well result in different sets of selected features. Furthermore, findings from this single-institution study have to be further tested and validated on external cohorts of patients, preferably in the setting of multi-center investigations, which are currently being planned to overcome the above-mentioned drawbacks.

## 5. Conclusions

AI-enhanced simultaneous ^18^F-FDG PET/MRI can non-invasively identify TNBC, the most aggressive tumor type requiring intensified treatment, with high accuracy. Additional investigations on larger cohorts of patients are necessary to validate our model and fully assess its generalizability.

## Figures and Tables

**Table 1:** Details of the developed radiomics model, including finally selected quantitative parameters and/or radiomic features.

Radiomic Model	PET/MR Images	Selected Features/Quantitative Parameters
***Quantitative parameters***	**DCE, ADC, PET parameters (Model 1)**	SUVmax, PF, ADCmean contralateral breast, ADCmean tumor lesion, MTT
***Radiomic features extracted from single ^18^F-FDG PET/MR images***	**ADCr** **(Model 2)**	cluster shade (GLCM)
		strength (NGTDM)
		Hdlge, hgce (NGLDM)
		hglze (SZM)
	**DCE** **(Model 3)**	kurtosis, coefficient of dispersion (FO)
		strength (NGTDM)
		joint maximum (GLCM)
	**PET** **(Model 4)**	glv, lglze (SZM)
		complexity (NGTDM)
		inverse difference moment (GLCM)
		rlv (RLM)
	**T2-w** **(Model 5)**	coefficient of variation (FO)
		entropy (NGLDM)
		run emphasis (RLM)
		gln (SZM)
***Combinations of radiomic features***	**ADCr, DCE** **(Model 6)**	auto correlation, cluster shade (GLCM, DCE)
		szhgle (SZM, DCE)
		sre (RLM, ADC)
		strength (NGTDM, DCE)
	**ADCr, DCE, PET** **(Model 7)**	zln (SZM, ADC)
		glv (SZM, PET)
		dcnNorm (NGLDM, PET)
		coefficient of variation, entropy (FO, PET)
***Integrated model of radiomic features and quantitative parameters***	**ADCr, DCE, PET + quantitative parameters** **(Model 8)**	SUVmax
		complexity (NGTDM, PET)
		inverse difference moment (GLCM, PET)
		minimum (FO, T2)
		kurtosis (FO, DCE)

**Note:** ADCr = radiomic features extracted from ADC maps; ADCmean = apparent diffusion coefficient mean of breast lesions; PF = plasma flow; MTT = mean transit time; DCE = radiomic features extracted from dynamic contrast-enhanced images; PET = radiomic features extracted from positron emission tomography images; T2-w = radiomic features extracted from T2-weighted images; SUV = standard uptake value; FO = first order parameter; GLCM = gray level cooccurrence matrix-based parameter; NGLDM = neighborhood gray level dependence matrix-based parameter; NGTDM = neighborhood gray tone difference matrix-based parameter; RLM = run length matrix-based parameter; SZM = size zone matrix-based parameter; glv = gray level variance; hgce = high gray level count emphasis; lzlgle = large zone low gray level emphasis; rln = run length non-uniformity; szlgle = small zone low gray level emphasis; zln = zone size non-uniformity. A full description of radiomics feature is reported in Supplementary Materials S3.

**Table 2:** Diagnostic accuracy of the eight developed radiomic models.

Model	Sensitivity	Specificity	PPV	NPV	Accuracy	AUROC
**1** **(Quantitative parameters)**	87.2 (82.4–90.5)	77.5 (72.6–82.2)	79.7 (76.2–83.5)	85.7 (81.2–89.2)	82.4 (78.9–85.7)	0.884 0.867–0.898)
**2** **(ADC)**	75.0 (67.6–81.1)	80.6 (75.3–84.9)	79.7 (74.6–84.1)	76.1 (70.7–80.8)	77.7 (72.8–81.6)	0.826 (0.789–0.857)
**3** **(DCE-derived RF)**	70.2 (62.2–77.0)	79.3 (72.6–84.9)	77.5 (72.2–82.6)	72.5 (67.1–77.2)	74.7 (70.1–78.9)	0.771 (0.720–0.814)
**4** **(PET-derived RF)**	68.4 (59.5–77.0)	75.9 (68.5–82.2)	74.2 (68.1–80.0)	70.4 (64.2–76.7)	72.1 (66.6–77.6)	0.789 (0.733–0.841)
**5** **(T2-derived RF)**	69.0 (62.1–74.3)	73.5 (68.5–78.1)	72.5 (68.1–76.5)	70.1 (65.0–74.7)	71.2 (66.7–75.5)	0.725 0.679–0.765)
**6** **(ADC, DCE-derived RF)**	83.7 (79.7–86.5)	67.4 (63.0–71.2)	72.3 (69.7–75.0)	80.3 (75.8–83.6)	75.6 (72.8–78.2)	0.822 (0.797–0.842)
**7** **(ADC, DCE, PET-derived RF)**	79.7 (71.6–86.5)	86.0 (80.8–90.4)	85.3 (80.9–89.4)	80.8 (75.0–86.3)	82.8 (78.2–87.1)	0.887 0.847–0.916)
**8** **(Radiomics features + quantitative parameters)**	88.9 (85.1–91.9)	74.4 (69.9–78.1)	77.9 (74.7–81.0)	86.9 (82.5–90.5)	81.7 (78.2–85.0)	0.871 (0.849–0.889)

**Note:** PPV = positive predictive value; NPV = negative predictive value; AUROC = area under the receiver operating characteristic curve; RF = radiomics features. Data in parentheses refer to 95% confidence intervals.

## Data Availability

The data presented in this study are available on request from the corresponding author.

## Supplementary Materials

The following supporting information can be downloaded at: https://www.mdpi.com/article/10.3390/cancers14163944/s1, S1: ^18^F-FDG PET/MRI acquisition protocol. S2: DWI and DCE image analysis. S3: Radiomic features extracted. S4: Histological features of included breast lesions. S5: Mean values and standard deviation (in parenthesis) of tumor size and quantitative parameters of triple negative and non-triple negative breast cancer lesions. S6: Comparisons in terms of diagnostic performance among the eight radiomics models according to the McNemar’s test. S7: Univariable analysis (Mann–Whitney test) for the comparison of quantitative parameters and radiomic features between triple negative and other breast cancer subtypes (in bold, significant *p* values < 0.05).
