# Supplementary material for: A Simultaneous Multiparametric 18F-FDG PET/MRI Radiomics Model for the Diagnosis of Triple Negative Breast Cancer
Source: Cancers (Basel). 2022 Aug 16;14(16):3944. doi: 10.3390/cancers14163944 (PMC9406327; doi:10.3390/cancers14163944)
Supplement: Supplementary file 1 [file cancers-14-03944-s001.zip › cancers-1822644-supplementary.pdf]

Supplementary Materials

S1. ^{18}F-FDG PET/MRI acquisition protocol

Patients were required to fast for five hours prior to the imaging examination. Each patient's blood glucose level was checked to ensure that it was < 150 mg/mg/dL, and then body weight-adjusted (200–350 MBq/kg body weight) ^{18}F-FDG was intravenously administered. PET/MRI acquisition started after an uptake time of 60 min. Attenuation correction was performed using the standard Dixon-based attenuation correction method [1, 2]. A 3D acquisition technique was employed with an axial and transverse field of view (FOV) of around 26 and 59 cm, respectively, and a sensitivity of 13.2 cps/kBq. Ordinary Poisson 3D ordered subset expectation maximization (OP-OSEM) (with Gaussian scatter correction) was used for the reconstruction of static PET images using 3 iterations and 21 subsets to produce a 172- image matrix 1.0 zoom including all standard corrections (normalization, scatter, random coincidences, and decay).

The acquisition of PET data lasted 30 minutes. MRI of the breast was performed using a dedicated 16-channel breast coil (Rapid Biomedical, Germany) as previously described [3], with the following sequences: morphological T2-weighted sequence; diffusion tensor imaging (DTI) single-shot spin-echo-prepared echo-planar imaging (EPI) sequence with parallel imaging and fat suppression, twelve directions, and two b-values (0 and 800); and high temporal resolution (16.7 s) T1-weighted Time-resolved angiography WIth Stochastic Trajectories (TWIST) dynamic contrast-enhanced (DCE) sequence, preceded by five pre-contrast gradient echo sequences with variable flip angles (2°, 10°, 20°, 30°, and 40°). Following an update to the breast MRI protocol (December 2017), high temporal resolution (14 s) T1-weighted TWIST Dixon dynamic sequence with 23 measurements was acquired as DCE sequence. DCE images were obtained before and after intravenous injection of macrocycle-structured gadolinium-based contrast agent (Gadoteric acid

Dotarem; Guerbet, Aulnay-sous-Bois, France) at a dose of 0.2 ml/kg body weight and a flow rate of 3.5 ml/s. Details of the breast MRI protocol are reported in **Table S1**.

Table S1. Details of the breast MRI acquisition protocol.

**Sequence**	**Acquisition plane**	**TR (ms)**	**TE (ms)**	**Matrix size**	**FOV (mm)**	**Slice thickness (mm)**	**Gap (mm)**	**Flip angle**
**T2-weighted**	Axial	4820	192	640×480	360×360	2.5	3	128°
**DTI**	Axial	4500	87	190×112	212×360	4	5.2	90°
**DCE – TWIST**	Axial	4.7	2.46	448×448	340×340	2	0	15°
**DCE – Dixon***	Axial	4.7	1.3	352×352	440×440	2	0	10.5°

Note: DTI = diffusion tensor imaging; DCE = dynamic contrast-enhanced; TWIST = Time-resolved angiography WIth Stochastic Trajectories

*After MRI update

S2. DWI and DCE image analysis

Breast cancer (BC) lesions were first identified on DCE images. Then, using DCE images as a reference, a circular 2D region of interest (ROI) was placed within tumor lesions (darkest solid areas) on apparent diffusion coefficient (ADC) maps, as recommended by the European Society of Breast Imaging (EUSOBI) international breast DWI working group for ADCmean calculation [4]. ADCmean of the contralateral breast parenchyma was also measured. DCE images were then evaluated for the extraction of perfusion quantitative parameters, using an open-source MRI perfusion analysis tool as a Horos plug-in (UMMPerfusion), using a pixel-by-pixel deconvolution approach to quantify perfusion data [5]. In detail, the arterial input function was plotted by placing a 2D ROI within the right cardiac ventricle. Then, BC lesions were annotated by drawing a 2D ROI over tumor margins, excluding macroscopic areas of necrosis, hemorrhage, and neighboring and intralesional vessels. The first post-contrast timepoint in which tumor lesions were clearly appreciable was chosen for the extraction of perfusion-weighted imaging (PWI) parameters including mean transit time (MTT), plasma flow (PF), and volume distribution (VD), according to previous evidence [6].

S3. Radiomic features extracted

First Order Parameters: These describe the grey level intensity distribution within the ROI. No account is taken of the spatial relationships between pixels.

min – minimum intensity
max – maximum intensity
mean – mean intensity
range – range of intensity values
std – standard deviation of intensity values
var – variance of intensity values (std squared)
median – median of intensity values
skewness
kurtosis
entropy
rms – root mean square
energy
totalEnergy
meanAbsDev – mean absolute deviation
medianAbsDev – median absolute deviation
P10 – 10th percentile intensity value
P90 – 90th percentile intensity value
robustMeanAbsDev – robust mean absolute deviation
robustMedianAbsDev – robust median absolute deviation
interQuartileRange
coeffDispersion – coefficient of dispersion
coeffVariation – coefficient of variation

Grey Level Cooccurrence Matrices-Based Parameters: These describe the relationships between pixels that are directly next to each other in the image.

energy
jointEntropy
jointMax
jointAvg – joint average
jointVar – joint variance
contrast
invDiffMom – inverse difference moment
invDiffMomNorm – inverse difference moment normalized
invDiff – inverse difference
invDiffNorm – inverse difference normalized
invar – invariance
dissimilarity
diffEntropy – difference entropy
diffVar – difference variance
diffAvg – difference average
sumAvg – sum average
sumVar – sum variance
sumEntropy
corr – correlation

clustTendency – cluster tendency
clustShade – cluster shade
clustPromin – cluster prominence
haralickCorr – Haralick correlation
autoCorr – auto correlation
firstInfCorr – information measure of correlation 1
secondInfCorr – information measure of correlation 2

Run Length Matrices-Based Features: These describe the extent of runs of pixels in a straight line with the same intensity.

gln – grey level non-uniformity
glnNorm – grey level non-uniformity normalized
glv – grey level variance
hglre – high grey level run emphasis
lglre – low grey level run emphasis
lre – long run emphasis
lrhgle – long run high grey level emphasis
lrlgle – long run low grey level emphasis
re – run emphasis
rln – run length non-uniformity
rlnNorm – run length non-uniformity normalized
rlv – run length variance
rp – run percentage
sre – short run emphasis
srhgle – short run high grey level emphasis
srlgle – short run low grey level emphasis

Size Zone Matrices-Based Parameters: These describe the extent of areas of pixels with the same intensity that are connected.

sze – small zone emphasis
lze – large zone emphasis
gln – grey level non-uniformity
glnNorm – grey level non-uniformity normalized
zln – size zone non-uniformity
zlnNorm – size zone non-uniformity normalized
zp – zone percentage
lglze – low grey large zone emphasis
hglze – high grey large zone emphasis
szlgle – small zone low grey level emphasis
szhgle – small zone high grey level emphasis
lzlgle – large zone low grey level emphasis
lzhgle – large zone high grey level emphasis
glv – grey level variance
zlv – size zone variance
ze – zone emphasis

Neighborhood Gray Level Difference Matrices-Based Parameters: These describe features related to the difference in intensity between a pixel and all of its neighbors.

lde – low dependence emphasis
hde – high dependence emphasis
lgce – low grey level count emphasis
hgce – high grey level count emphasis
ldlge – low dependence low grey emphasis
ldhge – low dependence high grey emphasis
hdlge – high dependence low grey emphasis
hdhge – high dependence high grey emphasis
gln – grey level non-uniformity
glnNorm – grey level non-uniformity normalized
dcn – dependence count non-uniformity
dcnNorm – dependence count non-uniformity normalized
dcp – dependence count percentage
glv – grey level variance
dcv – dependence count entropy
entropy
energy

Neighborhood Grey Tone Differences Matrices-Based Parameters:

coarseness
contrast
busyness
complexity
strength

S4. Histological features of included breast lesions

Histological type	Number of lesions	%
DCIS	3	3
IDC	79	80
ILC	7	8
IDC+ILC	3	3
Invasive micropapillary carcinoma	1	1
Invasive tubular carcinoma	1	1
Metaplastic carcinoma	1	1
Mucinous carcinoma	1	1
Papillary carcinoma	1	1
Apocrine carcinoma	1	1
Total	98	100
**Molecular subtype**	**Number of lesions**	**%**
Luminal A	10	10
Luminal B	51	52
HER2+	12	12
Triple negative	25	26
Total	98	100
**Tumor grade**	**Number of lesions**	**%**
G1	7	8
G2	35	35
G3	56	57
Total	98	100

Note: DCIS = ductal carcinoma in situ; IDC = invasive ductal carcinoma; ILC = invasive lobular carcinoma; HER2 = human epidermal growth factor receptor 2

S5. Mean values and standard deviation (in parenthesis) of tumor size and quantitative parameters of triple negative and non-triple negative breast cancer lesions.

	**TNBC**	**Non-TNBC**	***p value***
**Size**	31.4 (12.7)	27.3 (15.5)	0.506
**ADC_Mean**	0.860 (0.152)	0.942 (0.261)	0.199
**ADC_Mean_cbp**	1.921 (0.301)	1.965 (0.309)	0.536
**PF**	63.25 (26.12)	68.21 (38.52)	0.867
**VD**	70.17 (26.18)	75.17 (26.18)	0.772
**MTT**	74.79 (21.44)	76.92 (25.39)	0.708
**SUVmax**	9.5 (5.2)	4.9 (3.2)	<0.001*
**SUVmean**	5.7 (2.6)	3.3 (1.7)	<0.001*
**SUVmin**	3.1 (1.2)	2.0 (0.7)	<0.001*
**SUVmean_ibp**	1.1 (0.4)	1.0 (0.4)	0.327
**SUVmean_cbp**	0.9 (0.4)	0.9 (0.4)	0.908

Note: TNBC = triple negative breast cancer: ADCmean = apparent diffusion coefficient mean of breast lesions; PF = plasma flow; VD = volume distribution; MTT = mean transit time; SUVmax = maximum standard uptake value of breast lesions; SUVmean = mean standard uptake value of breast lesions; SUVmin = minimum standard uptake value of breast lesions; SUVmean_ibp = mean standard uptake value of ipsilateral breast parenchyma; SUVmean_cbp **=** mean standard uptake value of contralateral breast parenchyma; *Significant *p* values (≤ 0.005).

S6. Comparisons in terms of diagnostic performance among the eight radiomics models according to the McNemar's test

	**ADCr (Model 2)**	**DCE (Model 3)**	**PET (Model 4)**	**T2-w (Model 5)**	**ADCr/DCE (Model 6)**	**ADCr/DCE/PET (Model 7)**	**DCE, ADC, PET quantitative parameters (Model 1)**
**DCE (Model 3)**	0.720						
**PET (Model 4)**	0.416	0.734					
**T2-w (Model 5)**	0.356	0.653	1.000				
**ADCr/DCE (Model 6)**	0.810	1.000	0.649	0.571			
**ADCr/DCE/PET (Model 7)**	0.358	0.162	0.065	0.051*	0.200		
**DCE, ADC, PET quantitative parameters (Model 1)**	0.435	0.208	0.087	0.069	0.253	1.000	
**ADCr, DCE, PET + quantitative parameters (Model 8)**	0.519	0.261	0.115	0.092	0.314	0.890	1.000

Note: *Significant *p* values ($\leq$ 0.005); ADCr = radiomic features extracted from ADC maps; DCE = radiomic features extracted from dynamic contrast-enhanced images; PET = radiomic features extracted from positron emission tomography images; T2-w = radiomic features extracted from T2-weighted images.

S7. Univariable analysis (Mann–Whitney test) for the comparison of quantitative parameters and radiomic features between triple negative and other breast cancer subtypes (in bold, significant *p* values < 0.05)

	p value (Mann-Whitney test)
Quantitative Parameters	
SUV max	**< 0.001**
plasma flow	0.867
ADCmean (breast)	0.610
ADCmean (tumor)	0.199
MTT	0.804
ADC (Radiomics)	
cluster shade (GLCM)	**0.014**
strength (NGTDM)	0.060
hdlge (NGLDM)	0.016
hgce (NGLDM)	0.016
hglze (SZM)	0.017
sre (RLM)	0.239
zln (SZM)	0.051
DCE (Radiomics)	
kurtosis (FO)	**0.034**
coeffDispersion (FO)	0.282
strength (NGTDM)	0.129
joint max (GLCM)	0.137
auto correlation (GLCM)	0.723
cluster shade (GLCM)	0.342
szhgle (SZM)	0.729
PET (Radiomics)	
glv (SZM)	0.634
complexity (NGTDM)	0.593
lglze (SZM),	0.599
idm (GLCM)	0.208
rlv (RLM)	0.137
dcnNorm (NGLDM),	0.130
coeffVariation (FO)	0.057
entropy (FO)	0.146
T2 (Radiomics)	
coeffVariation (FO)	**0.009**
entropy (NGLDM),	**0.021**
run emphasis (RLM)	**0.035**
gln (SZM)	0.165
minimum (FO)	**0.008**

References

[1] Rausch, I.; Rust, P.; DiFranco, M.D.; Lassen, M.; Stadlbauer, A.; Mayerhoefer, M.E.; et al. Reproducibility of MRI Dixon-Based Attenuation Correction in Combined PET/MR with Applications for Lean Body Mass Estimation. J. Nucl. Med. **2016**, 57, 1096–1101. https://doi.org/10.2967/jnumed.115.168294

[2] Martinez-Möller, A.; Souvatzoglou, M.; Delso, G.; Bundschuh, R.A.; Chefd'hotel, C.; Ziegler, S.I.; et al. Tissue Classification as a Potential Approach for Attenuation Correction in Whole-Body PET/MRI: Evaluation with PET/CT Data. J. Nucl. Med. **2009**, 50, 520–526. https://doi.org/10.2967/jnumed.108.054726

[3] Romeo, V.; Clauser, P.; Rasul, S.; Kapetas, P.; Gibbs, P.; Baltzer, P.A.T.; et al. AI-enhanced simultaneous multiparametric 18F-FDG PET/MRI for accurate breast cancer diagnosis. Eur. J. Nucl. Med. Mol. Imaging **2022**, 49, 596–608. https://doi.org/10.1007/s00259-021-05492-z

[4] Baltzer, P.; Mann, R.M.; Iima, M.; Sigmund, E.E.; Clauser, P.; Gilbert, F.J.; et al. Diffusion-weighted imaging of the breast—a consensus and mission statement from the EUSOBI International Breast Diffusion-Weighted Imaging working group. Eur. Radiol. **2020**, 30, 1436–1450. https://doi.org/10.1007/s00330-019-06510-3

[5] Zöllner, F.G.; Weisser, G.; Reich, M.; Kaiser, S.; Schoenberg, S.O.; Sourbron, S.P.; et al. UMMPerfusion: an Open Source Software Tool Towards Quantitative MRI Perfusion Analysis in Clinical Routine. J. Digit. Imaging **2013**, 26, 344–352. https://doi.org/10.1007/s10278-012-9510-6

[6] Romeo, V.; Cavaliere, C.; Imbriaco, M.; Verde, F.; Petretta, M.; Franzese, M.; et al. Tumor segmentation analysis at different post-contrast time points: A possible source of variability of quantitative DCE-MRI parameters in locally advanced breast cancer. Eur. J. Radiol. **2020**, 126, 108907. https://doi.org/10.1016/j.ejrad.2020.108907
